# Atypical Neuropathic Symptoms Following Nexplanon: Possible Role of Edema-Induced Nerve Compression

**DOI:** 10.7759/cureus.88826

**Published:** 2025-07-26

**Authors:** Kelly Berk, Giorgiana Franzese, Ju Yong Koh

**Affiliations:** 1 Obstetrics and Gynecology, Midwestern University Chicago College of Osteopathic Medicine, Downers Grove, USA; 2 Obstetrics and Gynecology, Northwestern Medicine, Geneva, USA

**Keywords:** contraception, edema, nerve compression, neuropathic pain, nexplanon

## Abstract

Nexplanon is a subdermal contraceptive implant that is generally well tolerated; however, rare complications can occur. Although typical adverse events include localized pain and nerve irritation along specific dermatomes, diffuse neuropathic symptoms are uncommon. This case report suggests that subclinical edema-induced nerve compression, often accompanied by an inflammatory response, may contribute to such atypical presentations.

## Introduction

Nexplanon is a 2 mm × 4 cm subdermal implant containing 68 mg of etonogestrel, a synthetic progestin used for contraception. It is inserted in the upper non-dominant arm and remains in place for up to three years. Nexplanon works by secreting a synthetic progestin (etonogestrel), which is gradually absorbed into the subdermal capillary network and then distributed systemically via the bloodstream. The implant is made of ethylene vinyl acetate and contains inactive materials, such as barium sulfate, which confer radiopacity [1]. Complications associated with Nexplanon insertion and removal, as outlined in the prescribing information, include pain, paresthesia, bleeding, hematoma, scarring, infection, migration, and, more rarely, neuropathies. Although rare, upper extremity deep vein thrombosis (DVT) has also been reported. While large cohort studies, such as the Danish registry-based study, have not demonstrated a statistically significant association between the etonogestrel implant and venous thromboembolism (VTE) (relative risk 1.4; 95% confidence interval (CI), 0.6-3.4), the possibility of VTE cannot be entirely excluded, particularly in individuals with additional risk factors such as obesity, thrombophilia, tobacco use, or prolonged immobility [2]. Given these potential complications related to implant insertion and positioning, proper placement is critical to minimize risks such as device migration, nerve irritation causing paresthesia, vascular injury, or difficult removal.

The recommended insertion site is over the triceps muscle, approximately 8-10 cm from the medial epicondyle of the humerus and 3-5 cm posterior to the sulcus between the biceps and triceps. The implant is inserted in a distal-to-proximal direction to minimize the risk of injury to vascular and neural structures (e.g., brachial artery, basilic vein, ulnar and median nerves). Correct placement is confirmed by palpation of both ends of the implant post-insertion. Although Nexplanon insertion is generally well tolerated, rare complications such as peripheral neuropathies have been documented in fewer than 1% of cases [3]. 

Similar presentations to our case have been documented, including a likely allergic reaction to the barium sulfate in the device in a woman with a history of metal allergies, whose symptoms-such as edema and pain resolved only after removal of Nexplanon. In that case, however, pruritic lesions were also present, supporting the allergen hypothesis. In contrast, no such lesions were observed in our patient [4]. 

This case is unique because the patient’s pain and paresthesias were diffuse, not confined to a specific nerve distribution, and persisted despite confirmation of proper implant placement. In this case, the patient reported no relief with nonsteroidal anti-inflammatory drugs (NSAIDs) and acetaminophen. A tapered course of prednisone was subsequently trialed, resulting in only modest improvement. This contrasts with a previously reported case where radiating paresthesia and numbness following Nexplanon insertion was resolved entirely after two weeks of NSAIDs [5].

## Case presentation

An 18-year-old female with no significant past medical history presented to the emergency department (ED) in April 2024, with progressively worsening, constant pain and swelling of the left arm following Nexplanon replacement the previous day. Although she had experienced occasional paresthesias with her prior implant, she denied numbness or tingling at that time. With the new implant, however, she experienced a throbbing sensation, increased swelling, and severe pain that extended throughout the arm, accompanied by sensations of coldness and numbness upon movement.

**Figure 1:**
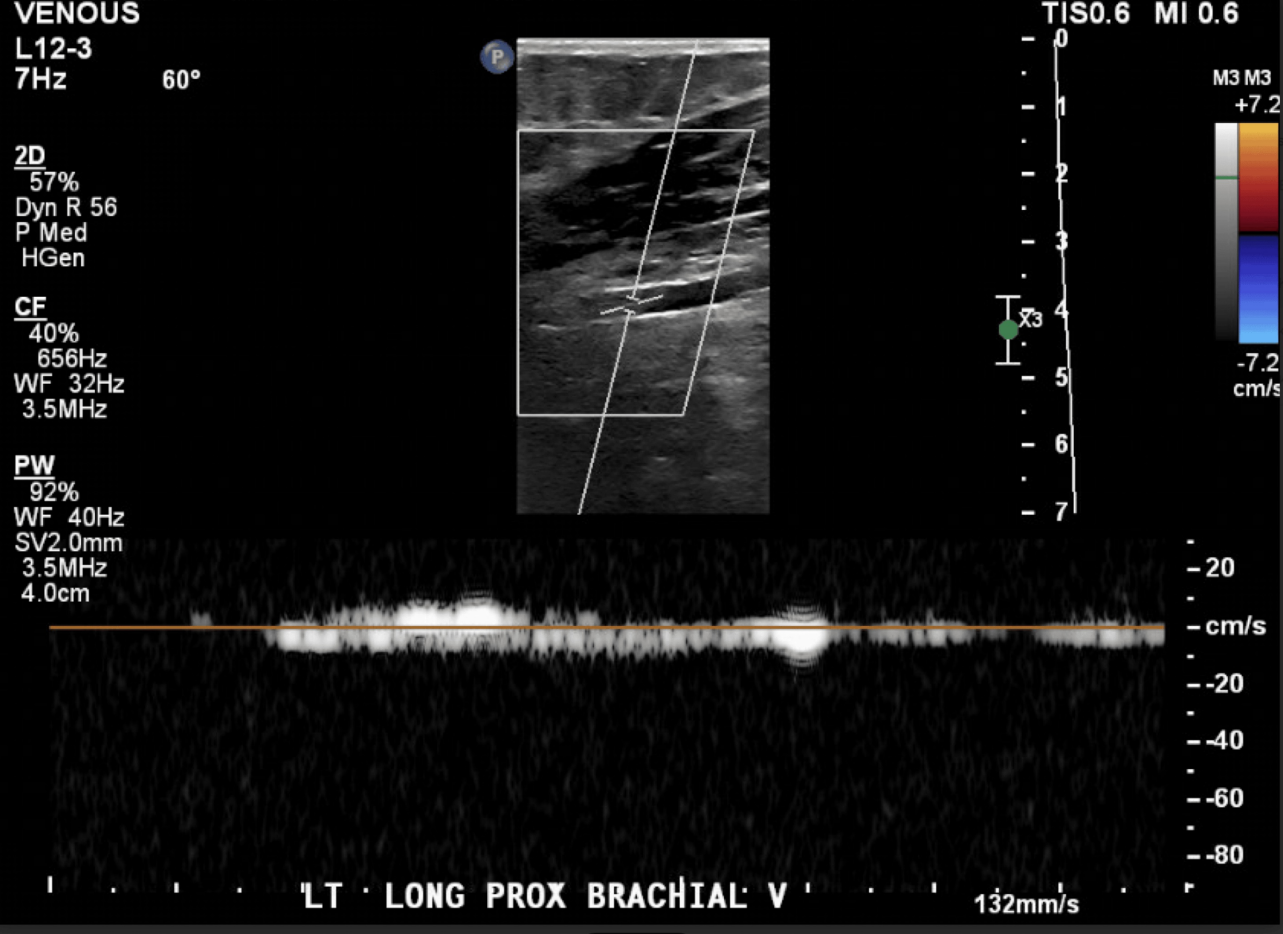
Spectral Doppler of the left proximal brachial vein demonstrating normal phasic venous flow without flow reversal, consistent with patent vessel and no evidence of thrombosis.

**Figure 2:**
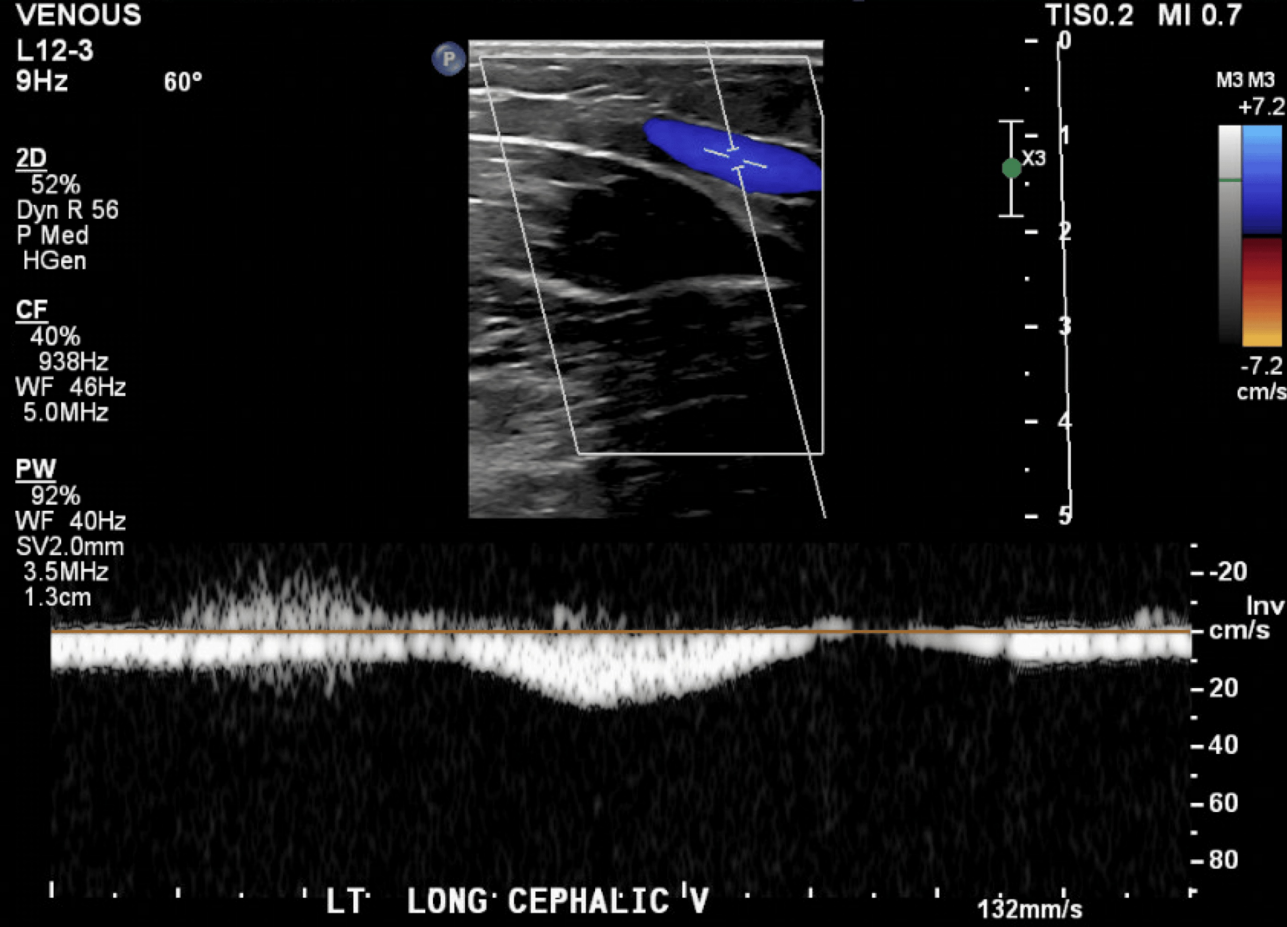
Color and spectral Doppler imaging of the left cephalic vein showing full vessel opacification with continuous, unimpeded flow. Findings are consistent with a patent vein and absence of thrombus.

**Figure 3:**
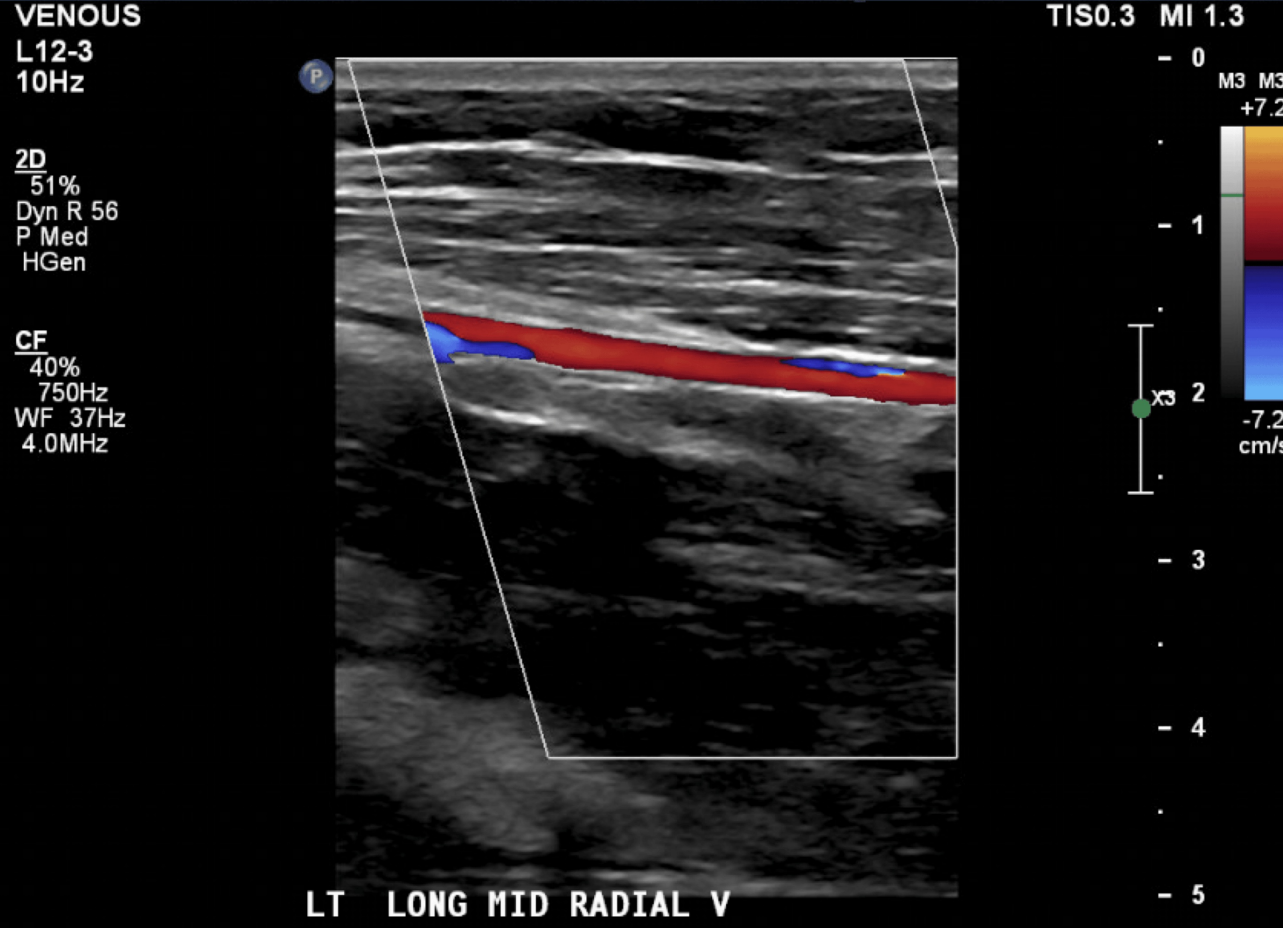
Color Doppler of the left mid radial vein with smooth, uninterrupted flow. No echogenic thrombus or intraluminal filling defects are identified, supporting exclusion of deep vein thrombosis (DVT).

On physical examination in the ED, the patient appeared well and non-toxic, with stable vital signs: blood pressure (BP) 144/90 mmHg (likely elevated due to acute pain), heart rate (HR) 80 beats per minute, temperature 98 °F, respiratory rate (RR) 16 breaths per minute, and SpO₂ 100% on room air. She exhibited moderate swelling localized to the left forearm, without overlying erythema or warmth, which suggested infection. There was ecchymosis at the Nexplanon insertion site, although no specific dimensions were documented. Two-point discrimination was not formally tested, but gross sensation was intact, and radial and ulnar pulses were present and strong. The arm was neurovascularly intact, with full passive range of motion at the shoulder, elbow, and wrist, although active movement was slow and limited by pain. Muscle strength was 5/5 throughout, though use was limited due to discomfort. No crepitus was noted, and there were no signs of infection. A venous duplex ultrasound revealed no evidence of DVT (Figures 1-3). The patient was managed with ibuprofen and acetaminophen and advised to follow up with her primary care physician (PCP).

Two months after insertion, the patient returned to her primary care provider, the same clinician who had placed the Nexplanon, reporting persistent discomfort, decreased sensation, and tingling in the left arm, which worsened with movement or palpation. Examination revealed slightly decreased grip strength and full passive range of motion at the shoulder, elbow, and wrist, although movement was slowed by pain. There was significant tenderness over the implant site. The implant remained correctly placed subcutaneously - approximately 10 cm above the medial epicondyle and 5 cm below the biceps notch. A lidocaine injection provided only minimal relief. An attempt at in-office removal was aborted due to severe pain, leading to referrals to general surgery and OB/GYN. 

The patient returned to the emergency department 11 days later with worsening numbness and tingling in all digits of the left hand. Examination revealed slightly decreased grip strength, full but slowed range of motion in the wrist, elbow, and shoulder, and marked tenderness over the implant site. A complete blood count with differential and a comprehensive metabolic panel were unremarkable, and repeat venous Doppler ultrasound showed no evidence of DVT. She was treated in the ED with Toradol and Solu-Medrol injections and discharged home with a prednisone taper and naproxen for continued symptom management.

Due to persistent symptoms affecting her daily functioning, she underwent surgical removal of the implant under anesthesia two months later. Intraoperative findings were unremarkable. Although steroid therapy provided partial symptom relief, the patient reported resolution of her symptoms only after complete removal of the Nexplanon implant.

## Discussion

Complications with implant insertions are rare, with an incidence of approximately 0.3% [3]. Most complications are self-resolving cases of ecchymosis, edema, and pain secondary to minor soft tissue trauma upon insertion and/or removal. Neural injury may occur, though it is usually secondary to deep insertion or implant migration after intramuscular or fascial placement (prescribing info). In this case, the patient developed diffuse, severe pain and swelling following Nexplanon insertion, despite normal laboratory and a normal venous Doppler ultrasound. Her symptoms were not confined to a single nerve distribution, and conventional treatments such as NSAIDs and acetaminophen provided little relief. A tapered course of prednisone yielded only modest improvement, suggesting that the underlying process was not completely mitigated by standard anti-inflammatory measures [5]. 

Nerve irritation may manifest as shooting, burning pain, and paresthesia localized to the affected nerve’s dermatome. For example, ulnar nerve irritation typically results in decreased sensation over the fourth and fifth digits, while median neuropathy presents as pain, numbness, and tingling affecting the palmar aspect of the thumb, index, middle, and radial half of the ring finger. Neuropathic presentations are usually secondary to nerve irritation or injury as evidenced by improper placement or scar tissue noted upon surgical removal, neither of which was grossly evident in this case [6]. Ulnar nerve injury is more often associated with implant removal but can occur during insertion due to improper technique, migration, or direct trauma, particularly in patients with minimal subcutaneous fat [7]. 

Injury to a specific nerve, most commonly the ulnar or median, has been documented with Nexplanon insertion and removal. These injuries typically present with a well-demarcated pattern of paresthesia along the corresponding dermatome, as previously described [8]. In contrast, this patient’s symptoms lacked a dermatomal pattern and did not respond to typical anti-inflammatory agents, raising the possibility of a more diffuse, non-focal mechanism of nerve irritation.

To explore this further, an understanding of neural microanatomy and the physiologic consequences of extrinsic compression is helpful. Peripheral nerves are encased in a layered sheath, comprising the endoneurium, perineurium, and epineurium, which protects both the axons and the microvascular network that nourishes them. The endoneurium, in particular, is permeable and plays a role in the repair of minor nerve injuries [9]. 

Studies have demonstrated that even modest extraneural pressures (approximately 4.0 kPa) can impair intraneural microvascular blood flow and disrupt axonal transport, leading to increased endoneurial fluid pressure and the development of subclinical edema [3,10]. Although such edema may not be visualized on standard imaging modalities (e.g., X-rays, ultrasound, or CT), and may fall below the resolution of conventional magnetic resonance imaging (MRI), it can increase intraneural pressure and impair nerve function. Advanced imaging, such as magnetic resonance (MR) neurography, may be necessary to detect subtle changes. This edema can provoke an inflammatory response characterized by endothelial thickening, fibroblast proliferation, and infiltration of inflammatory cells, which may compress adjacent neural structures and present clinically as diffuse neuropathic pain, paresthesias, and reactive swelling similar to what was observed in this case [11]. 

In our patient, the implant may have triggered a localized inflammatory response, leading to the development of subclinical edema. Although this subtle edema may not be readily detectable on standard imaging modalities such as X-rays or routine ultrasound, it can, nonetheless, intermittently compress nearby nerve fibers. The combined effects of mechanical compression from subclinical edema and the associated inflammatory cascade likely underlie the diffuse distribution of pain and paresthesia observed in this case. Additionally, the modest improvement with a steroid taper suggests an inflammatory process, while complete symptom resolution following device removal supports subclinical edema and nerve compression as the underlying cause.

In summary, the clinical picture in this case most strongly supports subclinical edema-induced nerve compression, augmented by an inflammatory response as the primary mechanism behind the patient’s diffuse neuropathic symptoms following Nexplanon insertion. Early recognition of this mechanism is essential for timely intervention, which, in this case, ultimately required implant removal to achieve symptom resolution and restoration of normal function.

## Conclusions

Educating both clinicians and patients on the potential complications associated with subcutaneous contraceptive implants is essential. Although serious adverse events remain rare, awareness of issues such as subclinical edema-induced nerve compression can lead to earlier recognition and intervention. Healthcare providers must adhere to optimal insertion techniques, ensuring proper anatomical placement and depth, to minimize the risk of nerve compression and other complications.
